# Efficient Generation of P53 Biallelic Mutations in Diannan Miniature Pigs Using RNA-Guided Base Editing

**DOI:** 10.3390/life11121417

**Published:** 2021-12-17

**Authors:** Honghui Li, Wenmin Cheng, Bowei Chen, Shaoxia Pu, Ninglin Fan, Xiaolin Zhang, Deling Jiao, Dejia Shi, Jianxiong Guo, Zhuo Li, Yubo Qing, Baoyu Jia, Hong-Ye Zhao, Hong-Jiang Wei

**Affiliations:** 1 Yunnan Key Laboratory of Porcine Gene Editing and Xenotransplantation, Kunming 650201, China; lihonghui@ynau.edu.cn (H.L.); cheng_8097@163.com (W.C.); cbw2006111@gmail.com (B.C.); shaoxiapu@gmail.com (S.P.); funnyl571271512@163.com (N.F.); xiaolinzhang2019@163.com (X.Z.); jiaodeling@163.com (D.J.); shidj2021@126.com (D.S.); 18788541129@163.com (J.G.); zhuoli2018@163.com (Z.L.); qingyubo20@163.com (Y.Q.); jiabaoyu2009@163.com (B.J.); 2 Faculty of Animal Science and Technology, Yunnan Agricultural University, Kunming 650201, China; 3 College of Veterinary Medicine, Yunnan Agricultural University, Kunming 650201, China; 4 College of Plant Protection, Yunnan Agricultural University, Kunming 650201, China

**Keywords:** cancer, P53 gene, BE3 system, SCNT, point mutation

## Abstract

The base editing 3 (BE3) system, a single-base gene editing technology developed using CRISPR/Cas9n, has a broad range of applications for human disease model construction and gene therapy, as it is highly efficient, accurate, and non-destructive. P53 mutations are present in more than 50% of human malignancies. Due to the similarities between humans and pigs at the molecular level, pig models carrying P53 mutations can be used to research the mechanism of tumorigenesis and improve tumor diagnosis and treatment. According to pathogenic mutations of the human P53 gene at W146* and Q100*, sgRNAs were designed to target exon 4 and exon 5 of the porcine P53 gene. The target editing efficiencies of the two sgRNAs were 61.9% and 50.0%, respectively. The editing efficiency of the BE3 system was highest (about 60%) when C (or G) was at the 5th base. Puromycin screening revealed that 75.0% (21/28) and 68.7% (22/32) of cell colonies contained a P53 mutation at sgRNA-Exon5 and sgRNA-Exon4, respectively. The reconstructed embryos from sgRNA-Exon5-5# were transferred into six recipient gilts, all of which aborted. The reconstructed embryos from sgRNA-Exon4-7# were transferred into 6 recipient gilts, 3 of which became pregnant, resulting in 14 live and 3 dead piglets. Sequencing analyses of the target site confirmed 1 P53 monoallelic mutation and 16 biallelic mutations. The qPCR analysis showed that the P53 mRNA expression level was significantly decreased in different tissues of the P53 mutant piglets (*p* < 0.05). Additionally, confocal microscopy and western blot analysis revealed an absence of P53 expression in the P53 mutant fibroblasts, livers, and lung tissues. In conclusion, a porcine cancer model with a P53 point mutation can be obtained via the BE3 system and somatic cell nuclear transfer (SCNT).

## 1. Introduction

The base editing 3 (BE3) system is engineered to fuse the expression of cytidine deaminase and uracil glycosylase inhibitor protein using CRISPR/Cas9n. The system can achieve targeted C–T conversion through the sgRNA in cells. Since it does not require dsDNA backbone cleavage (DSB) or a donor template, the BE3 system is characterized by accurate and nondestructive editing [1]. At present, it has been successfully used to perform single-base editing in various plants and animals [2,3,4,5,6,7,8,9,10]. As single-base mutations are the main cause of many human genetic diseases [11], the BE3 system possesses remarkable technical advantages and great prospects in the construction of accurate human disease models and gene therapy. For instance, a base conversion at MSTN, Tyr, and Lmna was designed to generate a premature stop codon or RNA mis-splicing by using the BE3 system. These genes have been successfully mutated to generate rabbit models with albinism, Hutchinson–Gilford progeria syndrome (HGPS), and double-muscled phenotypes [6].

P53 is an important tumor suppressor gene. Genetic variations of this gene result in human cancers in different ways. It maintains a high level of cellular expression in tumor cells and about half of tumors have P53 mutations [12]. Moreover, in some of the most difficult-to-treat cancers, P53 is mutated in at least 80% of samples [13]. To better understand the mechanism of tumorigenesis caused by P53 mutations and to develop a method of treatment and prevention, animal models are feasible tumor research platforms. The P53 knockout mouse model produced by gene editing has become important for tumorigenesis research because the development and progression of multiple tumors in mice are homologous to the development and progression in human cancer patients [14]. In addition, alteration or silences of the P53 gene in tree shrews and canines can also be used as models for cancer research [15,16,17]. However, the disease’s heterogeneity is typically not observed in these models as in humans [18], and the organs and tumors are usually too small for some type of studies, such as intensity-modulated radiation therapy and high-resolution, intensity-modulated treatments [19]. Therefore, the construction of larger, longer-lived animal models that accurately mimic human cancer is expected to be an effective research tool.

Pigs are anatomically and physiologically analogous to humans. Constructed genetically modified pigs with key genes make them better biomedical models for studying human diseases than laboratory rodents [20], and these porcine models can play a remarkable role in a better understanding of the etiology of many human diseases and their potential treatments. Spontaneous cancers in pigs are as rare as in humans [21], and the similarities between humans and porcine models are conservative even at the molecular level in cancer biology [19]. While the use of pigs that are genetically modified by targeting cancer-specific genes holds promise for the replication of human cancers and the production of cancer models, previous efforts have experienced difficulties. For example, efforts to produce TP53 mutant piglets have produced heterozygous cells [22], exhibited genomic instability [23], or had extremely low gene-targeting efficiency [24,25]. TALENs and CRISPR/Cas9 can lead to many potential defects such as unexpected indels, off-target cleavage, and decreased cell proliferation because of a reliance on the generation of a DSB. However, this mutation is random and cannot precisely mimic human P53 gene mutation [26,27]. So, rather than relying on stochastic disruption of the gene, it is necessary to use accurate and non-destructive disease models and correct point mutation in the target locus for studying the root cause of the disease and carrying out gene therapy.

In this study, we established the P53 mutant fetal fibroblasts of Diannan miniature pigs via the BE3 system. Piglets with mutant P53 were produced by somatic cell nuclear transfer (SCNT). The efficiencies of base substitution and random-base indels were tested. We further identified the phenotypic characteristics of the mutant pigs and measured the off-target rate of the BE3 system. These point-mutated P53 Diannan miniature pigs can precisely mimic human P53 gene mutation and may provide a powerful new tool for research on the formation and metastasis of malignant tumors.

## 2. Materials and Methods

### 2.1. Animals and Chemicals

All chemicals were purchased from Sigma Chemical Co. (St. Louis, MO, USA) unless otherwise stated. All animal experiments were performed with approval from the Bioethics Committee of Yunnan Agricultural University in China.

### 2.2. sgRNA Design and Vector Construction

**Figure 1:**
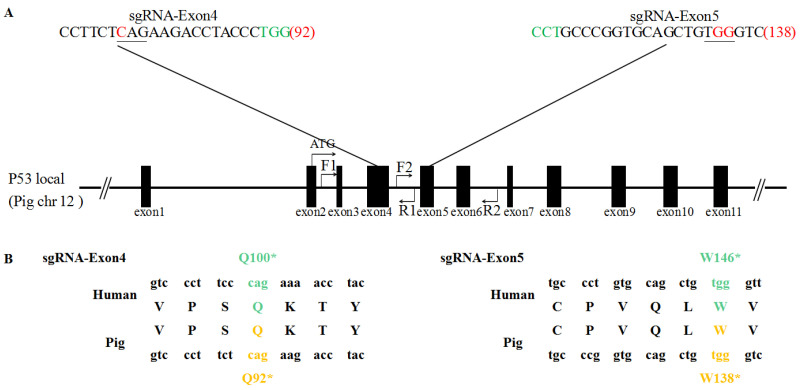
Conversion of C-to-T by the BE3 system. (**A**) Schematic diagram of the target site at the P53 locus. sgRNA sequences are presented in black. PAM sequences are highlighted in green. The BE3-mediated nucleotide substitutions are marked in red and underlined. (**B**) Diagrammatic representation of the mutations associated with P53 in humans and pigs.

The pCMV-BE3 plasmid was kindly provided by Professor Xingxu Huang (Shanghai University of science and technology, China). sgRNAs targeting exon 4 and exon 5 of the porcine P53 gene were designed manually, according to the principle of BE3 system base editing [1] and pathogenic mutations of the human P53 gene at W146* and Q100* (Figure 1A). The sgRNA expression vector pGL3-U6-sgRNA-PGK-Puromycin (Addgene plasmid #51133) was digested with BsaI and the linearized vector was gel purified. The pairs of oligos for the P53 targeting site (Table 1) were annealed, phosphorylated, and ligated to the vector. Finally, pGL3-U6-P53-sgRNA-Exon5 and pGL3-U6-P53-sgRNA-Exon4 were obtained.

### 2.3. Cell Culture, Transfection, and Selection

The preparation of pig fetal fibroblasts (PFFs) was performed as in our previous study [28]. Briefly, the PFFs were first thawed and then cultured in a medium containing 10% fetal bovine serum and 1% penicillin-streptomycin antibiotics. Once the cells attained 80% confluence, they were used for transfection. A pCMV-BE3 plasmid (10.5 μg) was taken and PFFs (~7 × 10^5^) in 700 μL PBS were mixed with the plasmid and the pGL3-U6-P53-sgRNA-Exon5/Exon4 plasmids (10.5 μg) were transfected by electroporation at 250 V for a single 20 ms pulse (Gene PulserXcell Microbial System, Bio-Rad, Hercules, CA, USA) in a 4mm gap cuvette. Then, the cells were seeded into 5 mL of fresh DMEM containing 10% FBS and 1 μg/mL puromycin in a T25 flask and incubated at 37 °C for 48 h. Cells were digested with 0.25% trypsin-EDTA for 2 m, then added to a complete culture medium to stop digestion, flowed by 1500 rpm centrifugation for 3 m, and the supernatant was pipetted out. Some of the cells were used for BE3 efficacy testing, and the remaining cells were cultured using an extremely dilute culture method, producing single-cell colonies. After 12-14 d, the colonies were assessed by PCR using primers (F1/R1:GGGAAGCACAGACCTATACTGACTC/ATGGAGAGCGAACAGAAGGTCAGAG; F2/R2:GACCCTGGTCCCAAAGTTGAATAC/GCAGGTCAAGTGAGAAGGAGAAAG), and the amplified fragments were then used for genotyping, including restriction endonuclease analysis and sequencing. Finally, we selected the positive fibroblast cell lines with biallelic mutations and used them as a donor for SCNT.

### 2.4. SCNT and Embryo Transfer

SCNT and embryo transfer were performed as described in our previous study [28]. First, oocytes were collected and cultured for in vitro maturation. Then, denuded oocytes were enucleated using a micromanipulator system. The donor cells were then injected into the perivitelline space of enucleated oocytes, and the reconstructed embryos were fused in an infusion medium using the Electro Cell Fusion Generator (LF201, Nepa Gene, Chiba, Japan) (200 V/mm for 20 μs) and activated in activation medium (150 V/mm for 100 μs). Subsequently, the reconstructed embryos were cultured in PZM-3 medium supplemented with 5 μg/mL cytochalasin B. The embryos were cultured for 2 h at 39 °C in a humidified atmosphere of 5% CO_2_, 5% O_2_, and 90% N_2_ and were then cultured in PZM-3 medium until embryo transfer.

In order to perform the embryo transfers, crossbred prepubertal gilts (large white/landrace duroc,) weighing 100–120 kg were selected as recipients for the reconstructed embryos. The reconstructed embryos were transferred surgically into the oviducts of surrogate gilts and 23 d later the gilts were diagnosed for pregnancy using an ultrasound scanner (HS-101 V, Honda electronics Co. Ltd., Toyohashi, Japan). After approximately 114 d, piglets were delivered by natural birth.

### 2.5. BE3 Efficiency Test

Porcine genomic DNA was extracted from ear samples of newborn piglets and partially screened cells by lysis buffer as described by Zhu et al. [29]. Using genomic DNA as a template, the primer pair mentioned above was used to amplify modified P53 alleles in screened cells by PCR, and the amplification products were subjected to T7E1 analysis or sequencing after purification using a gel extraction kit (28704, Qiagen, Valencia, CA, USA).

### 2.6. RNA Isolation and Quantitative PCR (qPCR)

Samples collected from P53 mutant piglets immediately after their death and WT piglets around 80 d of age were used for qPCR and immunoblotting. Samples were frozen immediately in liquid nitrogen and stored at −80 °C until use. The total RNA from liver, lung, kidney, heart, and brain tissues was isolated using TRIzol (15596018, Invitrogen, Carlsbad, CA, USA). cDNA was synthesized using a SuperScript RT Kit (RR047Q, TaKaRa Bio. Inc., Dalian, China) and used as a template in SYBR green-based qPCR (CFX-96, Bio-Rad, Hercules, CA, USA). Relative gene expression was analyzed by the 2^−ΔΔCT^ method after normalization to GAPDH mRNA abundance. The primer sequences are listed in Table 1.

### 2.7. Protein extraction and Immunoblotting

Protein extraction and immunoblotting were performed as described in our previous study [30]. PFFs were cultured at a density of 1 × 10^6^/well in a 10 cm plate and treated with or without DOX (100 μmol/L) for 24 h. P53 protein expression abundance was evaluated from the liver and lung tissue samples of WT and P53 mutant piglets and from the cells described above. Briefly, the liver and lung samples and cells were lysed using RIPA lysis buffer (P0013, Beyotime Biotechnology Co. Ltd., Shanghai, China) with protease inhibitors at 4 °C. After lysis, supernatants were obtained by centrifugation at 14,000× *g* for 15 m at 4 °C. Equal amounts (50 μg) of protein and a protein weight marker were separated by SDS-PAGE. After electrophoresis, the proteins were transferred to polyvinylidene difluoride (PVDF) membranes and reacted with primary antibodies against P53 (1:1000 *v/v*, ab32389, Abcam, Boston, MA, USA) and β-actin (1:5000 *v/v*, 22190126, Sigma, St. Louis, MO, USA). After incubation, membranes were washed and incubated with anti-rabbit secondary antibodies for P53 (1:5000 *v/v*, HAF008, R&D Systems, Minneapolis, MN, USA) and anti-mouse secondary antibodies for b-actin (HAF007, R&D Systems, Minneapolis, MN, USA). The membranes were developed using the ECL detection system (DW101-01, TransGen Biotech Co. Ltd., Beijing, China) and visualized with an imaging system (Universal Hood II, Bio-Rad, Hercules, CA, USA).

### 2.8. Immunohistochemical Analysis of Tissue Sections

The immunohistochemical analysis of the tissue sections was performed as reported previously [31]. In brief, the tissues were fixed using 4% paraformaldehyde at room temperature for 24 h and then transferred through a graded series of ethanol and xylene before being embedded in paraffin wax. Paraffin-embedded tissues were sectioned at a thickness of 5–8 μm. The sections were antigen-repaired with heat and blocked with endogenous peroxidase. The sections were then blocked by 5% bovine serum albumin in PBS for 15 m at 37 °C and incubated overnight in a humid chamber at 4 °C with an antibody against P53 (1:200 *v/v*, ab32389, Abcam, Boston, MA, USA) in blocking buffer. After 24 h, sections were washed with PBS three times and then incubated with a horseradish peroxidase (HRP)-conjugated secondary antibody (PV9000, ZSGB-BIO Ltd., Beijing, China). The sections were then counterstained with a DAPI staining solution and the slides were examined via microscopy (DM2000, Leica, Weltzlar, Germany).

### 2.9. Fluorescence Microscopy

PFFs (7.5 × 10^4^) were isolated from WT and P53 mutant piglets and cultured in a medium with or without 100 μmol/L DOX. After 24 h of culture, cells were fixed in 4% (*w/v*) paraformaldehyde overnight at 4 °C and permeabilized with 0.05% Triton X-100 for 30 m. Then, the cells were blocked with 1% bovine serum albumin in PBS for 1 h at room temperature and incubated with an antibody against P53 (1:200 *v/v*, ab32389, Abcam, Boston, MA, USA) at 4 °C overnight. The cells were washed three times with PBS, and then incubated with 400-fold diluted Alexa Fluor 488-labeled anti-mouse IgG (A28175, Invitrogen, Carlsbad, CA, USA). The nuclei were stained with 5μg/mL of DAPI, and the slides were covered with a mounting medium and observed under a laser scanning confocal microscope (FV1000, Olympus, Tokyo, Japan).

### 2.10. Off-target Assay

The genomic DNA of all newborn piglets was extracted to check for the possibility of off-target effects. Ten potential off-target sites (POTs) for sgRNA-Exon4 were predicted to analyze site-specific edits according to an online design tool (http://crispr.mit.edu/, accessed on 8 September 2018) [32]. The PCR products of the POTs were sequenced. All primers for the off-target assay are listed in Supplementary Data (Supplementary Material Table S1).

### 2.11. Statistical Analysis

All data were expressed as means ± standard deviations (SD). GraphPad Prism 8 software (GraphPad Software, La Jolla, CA, USA) was used to analyze all data using Student’s t-tests. Statistical significance was set at * *p* < 0.05, ** *p* < 0.01.

## 3. Results

### 3.1. Effective Mediation by the BE3 System of C-to-T Base Conversion in PFFs

**Figure 2:**
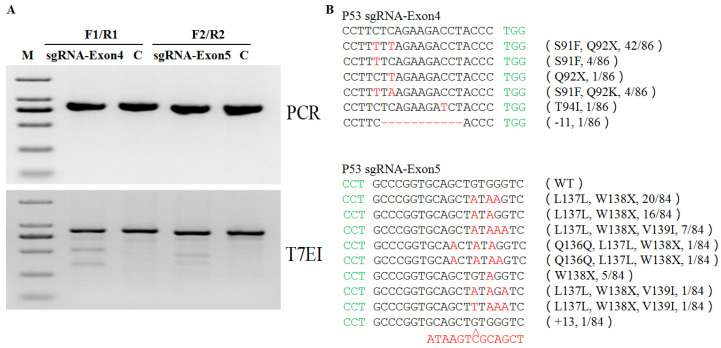
Targeting efficiency of P53-sgRNA-Exon4 and P53-sgRNA-Exon5. (**A**) Detection of sgRNA-Exon4 and sgRNA-Exon5: BE3-mediated base editing of P53 by PCR and T7EN1 cleavage assay. M, DNA marker; sgRNA-Exon4, P53sgRNA-Exon4; sgRNA-Exon5, P53sgRNA-Exon5; C, control. (**B)** Alignments of mutant sequences from targeted sequencing. PAM site and substitutions are shown in green and red, respectively. Relevant codons at the target site are underlined. The column on the right indicates amino acid mutation type, deletions (−), insertions (+), and proportion of positive colonies out of all sequenced colonies.

**Figure 3:**
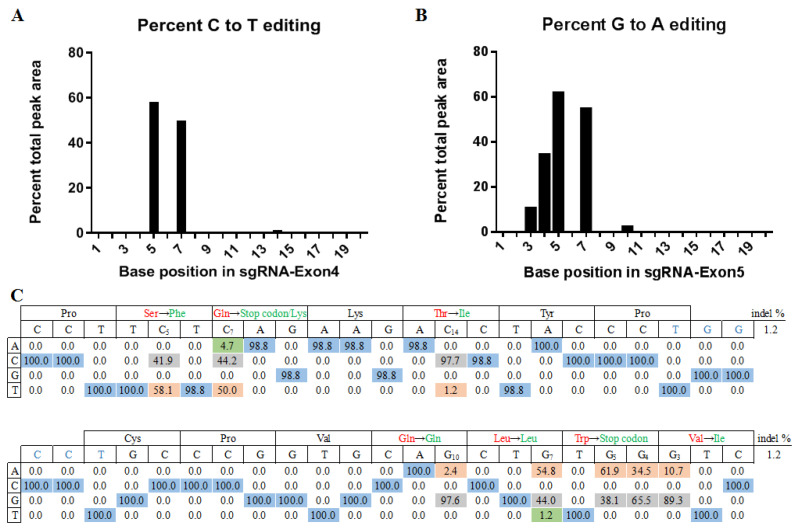
The predicted editing bar plot based on Sanger sequencing. (**A**) Base position in sgRNA-Exon4. (**B**) Base position in sgRNA-Exon5. (**C**) Synthetic 86-mers with sequences matching P53 gene sites were incubated with sgRNA-Exon4 and then analyzed for base editing by high-throughput DNA sequencing (HTS). Synthetic 84-mers with sequences matching P53 gene sites were incubated with sgRNA-Exon5 and then analyzed for base editing by HTS. The sequence of the protospacer is indicated to the right of the name of the site, with the PAM highlighted in blue. Underneath each sequence are the percentages of total DNA sequencing reads with the corresponding base.

To investigate whether the BE3 system can realize site-specific base conversion in the porcine genome, the sgRNAs were designed to target exon 5 and exon 4 of the porcine P53 gene (Figure 1). The C–T conversion by sgRNA-Exon5 in the P53 gene induced the desired W138* amino acid change, precisely mimicking the W146* mutation observed in humans (Figure 1B). The C–T conversion by sgRNA-Exon4 in the P53 gene induced the desired Q92* amino acid change, precisely mimicking the Q100* mutation observed in humans (Figure 1B). After T7E1 digestion, the WT had no band and both sgRNAs had two bands (Figure 2A). We observed 13 different base substitutions and 2 indels were observed at exon 5 and exon 4 (Figure 2B), including C-to-T and G-to-A conversions. Both sgRNAs were able to target the P53 gene and the target editing efficiencies of sgRNA-Exon5 and sgRNA-Exon4 were 61.9% and 50.0%, respectively (Table 2). The editing window of the BE3 system was from the 3rd base to the 7th base of the target sequence (the first base away from the PAM was one). The editing efficiency was highest (about 60%) when C (or G) was at the 5th base (Figure 3). In addition, a small number of unexpected mutations, such as random indels or non-C–T conversions were still produced in the BE3 editing system, but the efficiency was low, at only 2.9% and 1.2%, respectively (Table 2 and Figure 3).

### 3.2. Efficient Generation of P53 Mutant PFFs via the BE3 System

**Figure 4:**
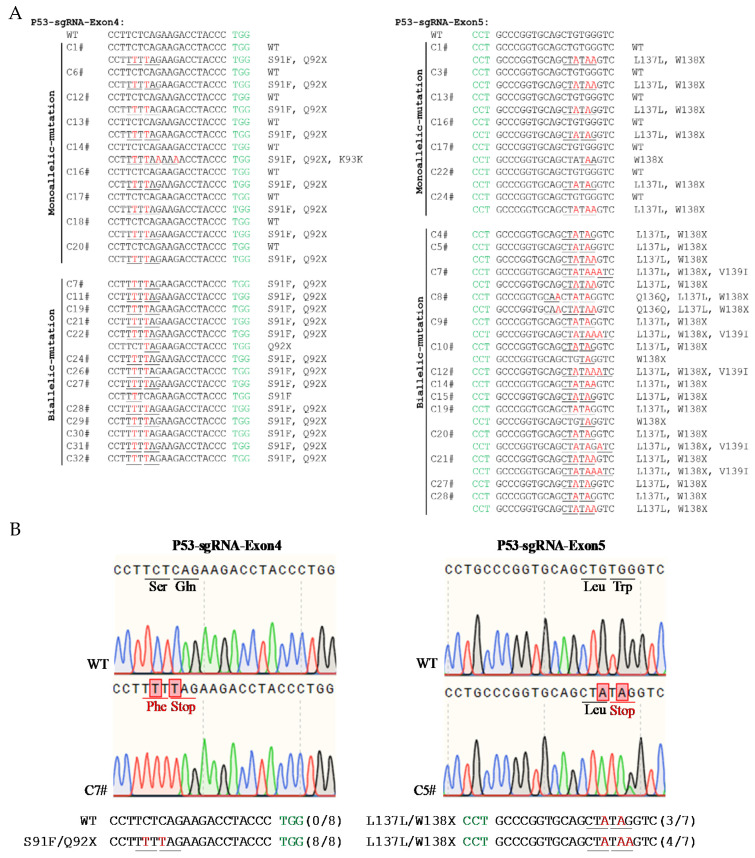
BE3-mediated mutations in the cells clones of P53-sgRNA-Exon4 and P53-sgRNA-Exon5. (**A**) The sequences of P53 mutant cell lines. The WT sequence is shown above. PAM site and substitutions are shown in green and red, respectively. Relevant codons at the target site are underlined. The column on the right indicates amino acid mutation type. (**B**) Representative sequencing chromatograms at the P53-sgRNA-Exon4 and P53-sgRNA-Exon5 targets of WT and edited pig cell clones. Target sequence (black), PAM region (green), target sites (red), mutant amino acids (underlined), and amino acid mutation types are indicated. The relevant codon identities at the target site are presented beneath the DNA sequence.

Porcine fetal fibroblasts were screened using puromycin and a dilution culture. We selected 28 and 32 single-cell colonies from P53-sgRNA-Exon5- and P53-sgRNA-Exon4-treated cells. As shown in Table 3, sgRNA-Exon5 generated 75.0% (7 monoallelic mutations and 14 biallelic mutations out of 28 colonies in total) and sgRNA-Exon4 generated 68.7% P53 mutation (9 monoallelic mutations and 13 biallelic mutations out of 32 colonies in total). Using TA cloning and sanger sequencing, we detected 35 P53 modifications in 56 alleles from 28 single-cell colonies, and all sgRNA-Exon5-induced P53 modifications contained a premature stop codon (TAA or TAG). sgRNA-Exon4 showed similar efficiency, yielding 35 P53 modifications in 64 alleles, with 33 of them harboring a missense mutation (TCT to TTT, Ser to Phe) and a premature stop codon (TAA or TAG), 1 having a premature stop codon (TAG) and 1 creating a missense mutation (TCT to TTT, Ser to Phe) (Figure 4).

### 3.3. Generation of P53 Mutant Piglets by SCNT

**Figure 5:**
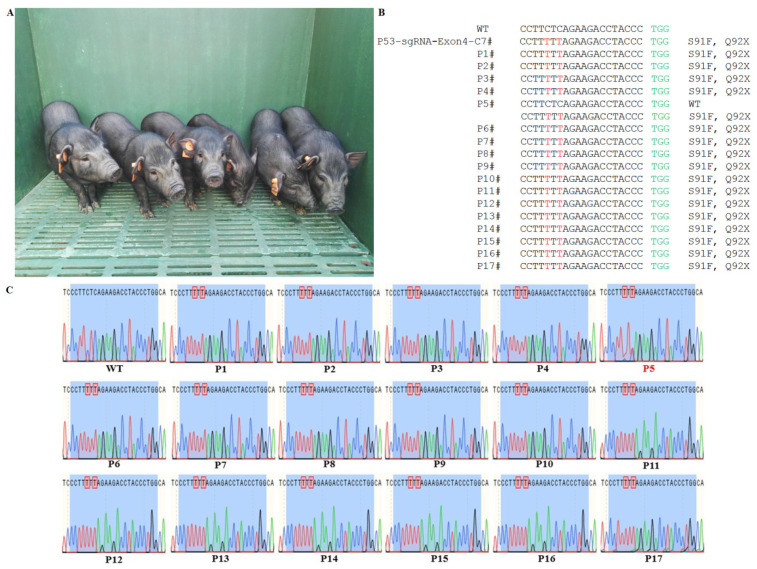
BE3-mediated P53 mutations in piglets. (**A**) The P53 mutant piglets (P1, P2, P6, P9, P12, and P15). (**B**) The WT sequence is shown above. PAM site and substitutions are shown in green and red, respectively. Relevant codons at the target site are underlined. The column on the right indicates the amino acid mutation type. (**C**) Sanger sequencing chromatograms of WT and mutant founder piglets. Red box indicates mutation. The piglets with monoallelic mutations are shown in red. WT: wild-type.

Cell colonies sgRNA-Exon5-5# and sgRNA-Exon4-7# were selected as donor cells for SCNT according to their growth status. The cleavage and blastocyst formation rates of the reconstructed embryos from sgRNA-Exon5-5# were 90.1% and 40.8%, respectively (Table 4). Additionally, 2130 reconstructed embryos were transferred into 6 recipient gilts, but all of them aborted. The cleavage and blastocyst formation rate of the reconstructed embryos from sgRNA-Exon4-7# were 81.0% and 37.9%, respectively. We transferred 1892 reconstructed embryos into 6 recipient gilts, 3 of which became pregnant, and obtained 14 live (Figure 5A) and 3 dead piglets (Table 5). Among the live piglets, 8 of them died before weaning (35d), 5 piglets died within 3 months after weaning, and 1 piglet survived up to 396 d (Table 6).

### 3.4. Functional Inactivation of the Porcine P53 Gene Mutation

**Figure 6:**
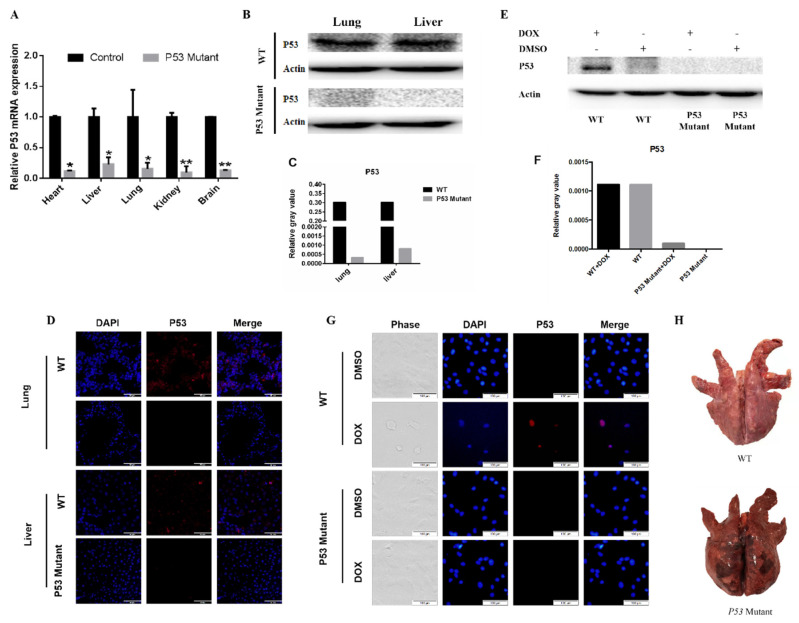
The detection of P53mRNA and proteins in piglets. (**A**) The relative expression levels of P53 mRNA in the different tissues from P53 mutant and WT piglets. (**B**) P53 protein expression in lung and liver. (**C**) Quantification of P53 protein expression in different organs in WT and P53 mutant pigs. β-actin was used as an internal control. (**D**) The intracellular localization of P53 was analyzed using fluorescence microscopy. The livers and lungs from P53 mutant piglets and WT piglets were stained with DAPI (blue) and an anti-P53 antibody (red). (**E**) The fibroblast cells were treated with DOX and DMSO and protein expression levels were examined by western blotting. (**F**) Quantification of P53 protein expression in different organs in fibroblast cells treated with DOX and DMSO. β-actin was used as an internal control. (**G**) The intracellular localization of P53 was analyzed using fluorescence microscopy. The fibroblast cells from P53 mutant piglets and WT piglets were treated with DOX for 24 h and stained with Hoechst 33,342 (blue) and an anti-P53 antibody (red). (**H**) Lung samples from a P53 mutant and a WT piglet after autopsy.

Based on the Sanger sequencing of all 17 piglets, each animal carried the base mutation at the target point. The targeting efficiency was 100%, including 1 monoallelic and 16 homozygous biallelic mutations (Figure 5B,C). The relative mRNA expression of P53 in the livers, hearts, lungs, brains, and kidneys of the P53 mutant piglets were significantly decreased compared to WT tissues (*p* < 0.05, Figure 6A). P53 was expressed in the liver and lung tissues of the WT porcine, but no detectable P53 expression was observed in the P53 mutant piglets (Figure 6B–D). In addition, doxorubicin (DOX) treatment resulted in robust P53 expression in WT fibroblasts. However, there was still no P53 expression in P53 mutant fibroblasts after DOX treatment (Figure 6E–G). Autopsy revealed wild-type piglets were normal but P53 mutant piglets exhibited severe pulmonary abnormalities (Figure 6H).

### 3.5. Off-Target Validation in Mutant Animals

To test whether off-target mutations occurred in these genetically modified piglets, we predicted 10 potential off-target sites by using an online design tool and designed the primers to detect off-target mutations in 17 piglets. The result showed that only one piglet (P5) had a detectable off-target mutation at two off-target sites (Supplementary Material Figure S1). The off-target rate was 5.9%.

## 4. Discussion

P53 is an important tumor suppressor; its mutation is one of the most frequent alterations in human cancers. Studies on P53 mutation can further contribute to the better understanding of P53 structure and function and have wide applications to cancer management and therapy [33]. Although murine and human P53 genes have highly homologous (80%) DNA sequences at the critical hotspot and post-translational modification sites, the intragenic suppressor sites differ [34]. In addition, current clinical imaging systems lack the capability to deal with the body size difference (approximately 1:1000) between mice and humans, limiting the application of murine models [35]. Pigs exhibit anatomical, physiological and pathophysiological similarities with humans. Through cDNA sequence comparison, the homology of P53 gene between pigs and humans is 83.84%, which is higher than that of mice and humans. Miniature pigs, in particular, are better-suited than small animals to serve as model animals for studying the optimization and validation of imaging technologies and surgical procedures. For example, Sieren et al. obtained a P53 mutant tumorigenesis model using Yucatan miniature pigs which recapitulated many aspects of human cancer development and supported the detection of tumors by using computed tomography (CT) and magnetic resonance imaging (MRI) technology [24].

The construction of large-animal disease models is time-consuming, expensive, and has a low success rate, which seriously restricts their development and application. Therefore, improving gene editing efficiency is extremely important. The BE3 system can induce targeted C:G to T:A conversions and can introduce numerous disease-relevant mutations into mammalian cells, resulting in a maximum base editing yield of 75% [1]. Liu et al. used BE3 to create targeted base substitutions in rabbits with a mutation efficiency about 75%~87% and an average successful target mutation rate of up to 70% [6]. In the current study, we designed a total of four sgRNAs and detected their editing activity by T7E1 restriction enzyme digestion. The results showed that the editing efficiency of sgRNA-Exon6-1 and sgRNA-Exon6-2 were lower (Supplementary Material Figure S2), so we chose sgRNA-Exon4 and sgRNA-Exon5 for subsequent experiments. The target mutation efficiencies of sgRNA-Exon5 and sgRNA-Exon4 were 61.9% and 50.0%, respectively. These results were lower than in the previous study [6], which may be due to the effect of different gene locations. However, the efficiency was significantly increased by using adeno-associated, virus-mediated homologous recombination and CRISPR/CAS9-mediated point mutation (1.1%, 4.7%) [24,36]. The results indicated that the BE3 technology does not have a high level of editing efficiency in all target sites because of certain limitations. The highly efficient target sites were obtained by the necessary screening.

In addition to efficiency, the precision of gene editing is also important for the construction of disease models in large animals. Although CRISPR/Cas9 has significantly improved gene mutation efficiency, it remains difficult to accurately mimic human pathogenic gene mutation because the DSBs produced by CRISPR/Cas9 lead to non-homologous end-joining (NHEJ) and exhibit randomness of gene editing [37]. The porcine P53 gene, located on chromosome 12, has 11 exons and is 87% conserved with human P53. In this study, we generated P53 mutations in exon 4 and exon 5 via the BE3 system. The C–T conversion by sgRNA-Exon4 or sgRNA-Exon5 in the P53 gene induced the desired Q92* or W138* amino acid changes, similar to the Q100* or W146* mutations observed in humans. Grünewald et al. analyzed all detected mutations of tumor samples according to the different human gene families and showed that c.298 C>T of exon 4 in the TP53 gene can induce the desired Q100* amino acid change [38]. Kuo et al. found four TP53 mutations in six human tumor samples, including c.437 G>A and c.706 T>C, which were novel [39]. Fésüs et al. also reported that c.298 C>T of exon 4 and c.437 G>A in the TP53 gene can induce the desired Q100* and W146* amino acid change [40]. Moreover, the nonsense mutation (c.437 G>A) has been classified as pathogenic [41]. These results further demonstrated that base editors can intervene with a highly efficient and precise single-amino acid change in the coding sequence of protein [1].

In this study, we used the BE3 system to modify P53 and screened positive fetal fibroblasts using puromycin and a dilution culture. After SCNT, we obtained 1 monoallelic and 16 homozygous biallelic mutant piglets. Among live P53 mutant pigs, P5# exhibited a heterozygous genotype, including the WT sequence, one missense mutation, and one nonsense mutation of sgRNA-Exon4. We considered that an off-target mutation happened in P5#. These findings were consistent with previous reports that the BE3 system can induce proximal off-targets, indels, and non-C-to-T conversions [1,4,42]. In our study, the average frequencies of these undesired mutation were 5.9%, 1.2%, and 2.9% while the average frequencies were 19%, 8%, and 3% in rabbit models, which may be due to the relatively wide editing window and may be reduced by effectively narrowing the deamination window and engineering cytidine deaminases to improve the precision of the base editors in the future [6].

We observed that the P53 mRNA expression levels in different tissues of the P53 mutant piglets were significantly decreased compared with the WT, and P53 protein expression was not detected in the P53 mutant piglets. We treated WT and P53 mutant fibroblasts with DOX and detected no subsequent P53 expression in the mutant fibroblasts. These results indicate that P53 was successfully knocked out. However, in line with previous research, we did not observe the tumor phenotypes of the P53 biallelic mutant pigs. It was reported that P53 biallelic knockout Diannan miniature pigs produced by SCNT did not manifest tumorigenic signs for <5 months [26]. Although P53 inactivation is sufficient for spontaneous tumorigenesis in pigs, animals younger than 16 months of age did not show any tumor phenotypes or other abnormalities [23], further confirming that long-term monitoring is required for tumor detection in P53 mutant pigs. Previously, it was reported that biallelic mutation pigs developed tumors after attaining puberty, while monoallelic mutation pigs fail to develop tumors [24]. The cause of fetal death could be the genomic instability, as hotspot mutations were reported to enhance metastasis and thus promote genomic instability that might contribute towards prenatal deaths [43]. Further, we suspected that the mutation site might be the cause of fetal mortality, but we could not find any evidence so there is need to explore these mechanisms. Most P53 knockout piglets developed lung inflammation, which may have been an early symptom of tumorigenesis.

## 5. Conclusions

Herein, we obtained P53 point mutant pigs via the BE3 system and somatic cell nuclear transfer. Our results indicated that the BE3 system can be used to precisely and efficiently construct porcine cancer models. These pigs will provide a powerful new resource for preclinical oncology and basic cancer research.

## Figures and Tables

**Table 1:** Primer information.

Primer Name	Sequences (5’→3’)
F1	GGGAAGCACAGACCTATACTGACTC
R1	ATGGAGAGCGAACAGAAGGTCAGAG
F2	GACCCTGGTCCCAAAGTTGAATAC
R2	GCAGGTCAAGTGAGAAGGAGAAAG
U6-F	CTCGACGGTATCGATCACGAGAC
P53-sgRNA-Exon4-F	ACCGCCTTCTCAGAAGACCTACCC
P53-sgRNA-Exon4-R	AAACGGGTAGGTCTTCTGAGAAGG
P53-sgRNA-Exon5-F	ACCGGACCCACAGCTGCACCGGGC
P53-sgRNA-Exon5-R	AAACGCCCGGTGCAGCTGTGGGTC
qP53-F	CACTGGATGGCGAGTATTTCAC
qP53-R	CGCAGTCTGGGCATCCTTC
qGAPDH-F	ATCAAGAAGGTGGTGAAGCAC
qGAPDH-R	CAGCATCAAAAGTGGAAGAGTG

**Table 2:** Summary of base editing efficiency using the BE3 system.

sgRNA	No. of Sequencing	Mutant Efficiency (%)				
		No. of Mutants	No. of Target Mutant	No. of Nontarget Mutant	No. of Indel	No. of non-C>T
*P53*-sgRNA-Exon5	84	53(63.1)	52(61.9)	1(1.2)	1(1.2)	1(1.2)
*P53*-sgRNA-Exon4	86	53(61.6)	43(50.0)	10(11.6)	1(1.2)	4(4.7)
Total	170	106(62.4)	94(55.3)	11(6.5)	2(1.2)	5(2.9)

**Table 3:** Colony-Targeting efficiency of P53-sgRNA-Exon5 and P53-sgRNA-Exon4.

Target	No. of Colonies	Monoallelic-Mutation (%)	Biallelic-Mutation (%)
*P53*-sgRNA-Exon5	28	7(25)	14(50)
*P53*-sgRNA-Exon4	32	9(28.1)	13(40.6)

**Table 4:** In-vitro developmental competence of the reconstructed embryos.

Donor Cells	No. of Reconstructed Embryos	Cleavage Rate (%)	Blastocyst Rate (%)
*P53*-sgRNA-Exon5-5	71	64(90.1)	29(40.8)
*P53*-sgRNA-Exon4-7	58	47(81.0)	22 (37.9)

**Table 5:** Production of P53 mutant piglets by SCNT.

Donor Cells	Recipients	Transferred Embryos	Days of Pregnancy (d)	Pregnancy Rate (%)	Offspring (Stillborn/Aborted)	Mutant Piglets
*P53*-sgRNA-Exon5-5	1	325	-	0	-	-
	2	325	-		-	-
	3	340	-		-	-
	4	340	-		-	-
	5	400	-		-	-
	6	400	-		-	-
Total	6	2130			0	0
*P53*-sgRNA-Exon4-7	1	355	-	50.0	-	
	2	307	-		-	
	3	300	117		3	3
	4	330	117		7 (1 dead)	7
	5	290	119		7 (2 dead)	7
	6	310	-		-	
Total	6	1892			14 (3 dead)	17

Note: - indicates no pregnancy.

**Table 6:** Summary of the P53 mutant piglets for their health status.

Piglet ID	Birth Weight (kg)	Survival Time
P1#	1.07	68 d
P2#	0.93	84 d
P3#	1.1	31 d
P4#	0.74	19 d
P5#	0.67	Stillborn
P6#	0.98	396 d
P7#	0.52	23 d
P8#	0.53	32 d
P9#	0.89	79 d
P10#	1.02	25 d
P11#	0.51	Stillborn
P12#	1.1	82 d
P13#	0.87	31 d
P14#	0.58	13 d
P15#	1.0	82 d
P16#	0.81	27 d
P17#	0.35	Stillborn

## Data Availability

Not applicable.

## Supplementary Materials

The following are available online at https://www.mdpi.com/article/10.3390/life11121417/s1, Figure S1: Off-target detection in piglets, Figure S2: (**A**) The information of sgRNAs design and the editing activity detected, (**B**) The information of all Western blot results, include densitometry readings/intensity ratio of each band, Table S1: The primers of potential off-target sites (POTS).
