# Supplementary material for: Efficient Generation of P53 Biallelic Mutations in Diannan Miniature Pigs Using RNA-Guided Base Editing
Source: Life (Basel). 2021 Dec 17;11(12):1417. doi: 10.3390/life11121417 (PMC8706133; doi:10.3390/life11121417)
Supplement: Supplementary file 1 [file life-11-01417-s001.zip › life-1474570_supplementary final.pdf]

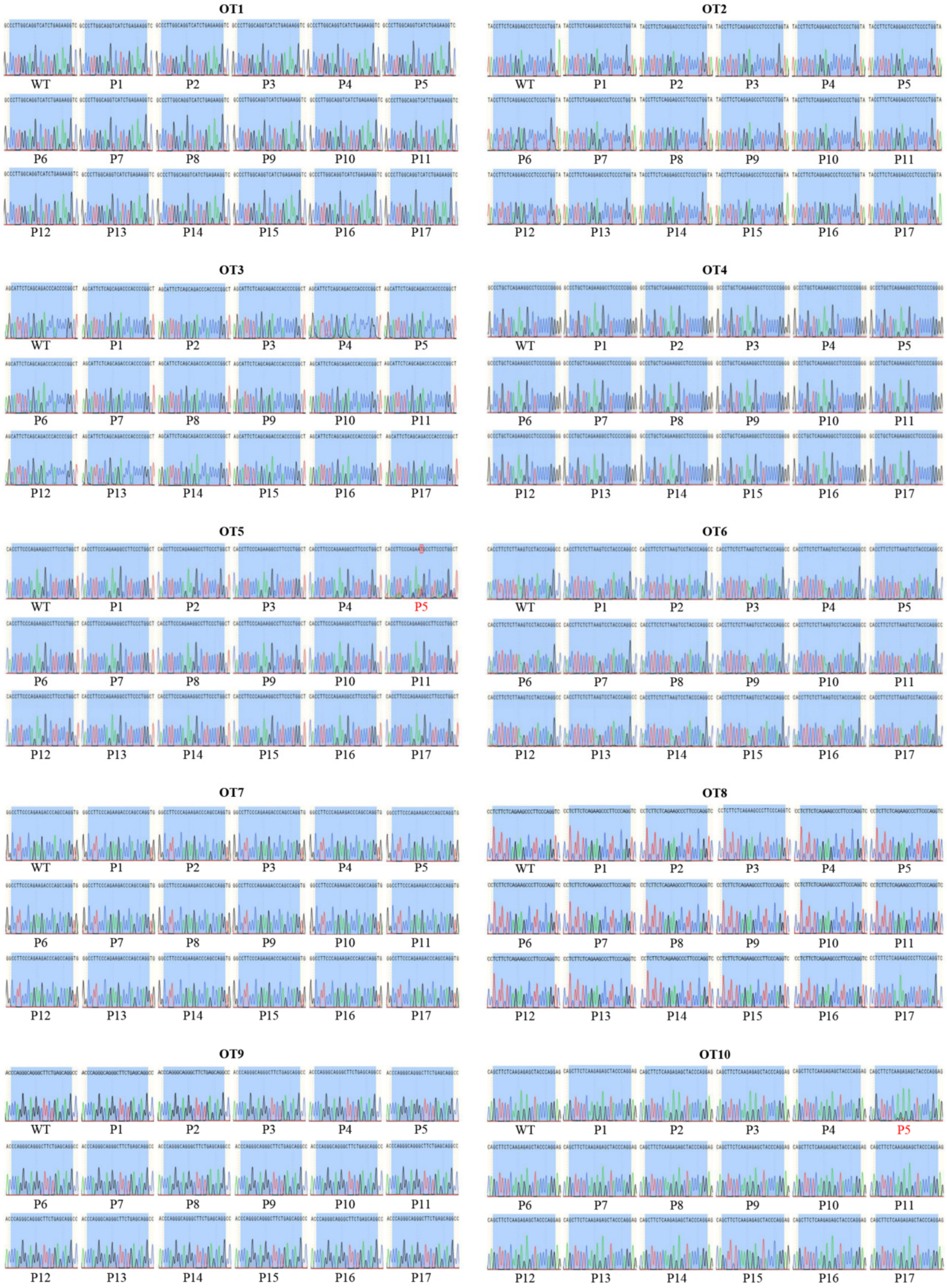

Figure S1. Off-target detection in piglets. Chromatogram sequence analysis of ten POTS for sgRNA using PCR products in P53. Red box labeled base indicates mutation. The piglets with off-target was shown in red. WT wild-type.

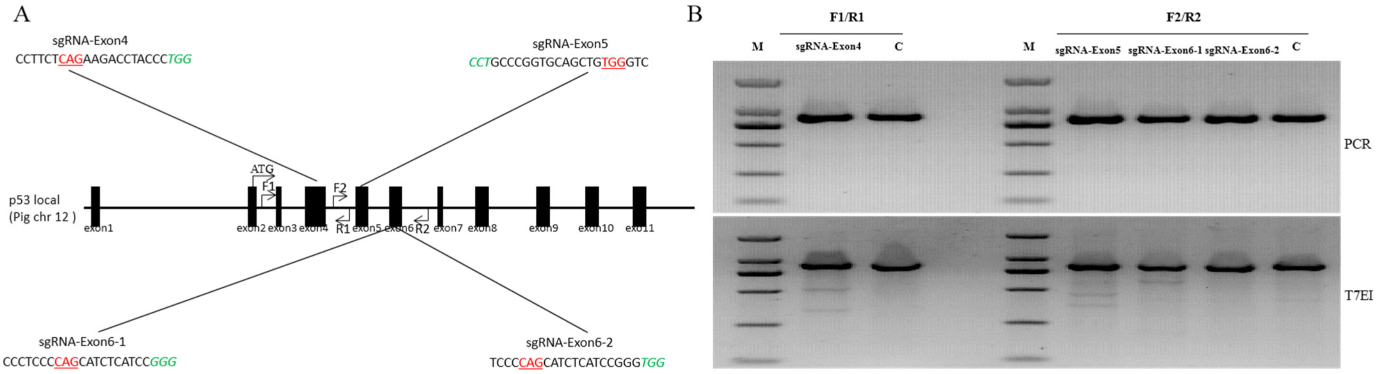

Figure S2. The information of sgRNAs design and the editing activity detected. (**A**) Schematic diagram of the target site at the P53 locus. sgRNA sequences are presented in black. PAM sequences are highlighted in green. The BE3-mediated nucleotide substitutions are marked in red and underlined. (**B**) Detection of sgRNA-Exon4, sgRNA-Exon5, sgRNA-Exon6-1 and sgRNA-Exon6-2: BE3-mediated base editing of P53 by PCR and T7EN1 cleavage assay. M, DNA marker; sgRNA-Exon4, P53sgRNA-Exon4; sgRNA-Exon5, P53sgRNA-Exon5; sgRNA-Exon6-1, P53sgRNA-Exon6-1; sgRNA-Exon6-2, P53sgRNA-Exon6-2; C, control.

Table S1. The primers of potential off-target sites (POTS).

NO.	Target	Position	Mismatches	Primer sequence
OT1	crRNA: CCTTCTCAGAAGACCTACCCNGG<br>DNA: CCTTCTCAGATGACCTGCCAAGG	chr1:191778281-191778303	3	F: GCTACAAGGAGCGTTAACTCAG<br>R: AGTTCATAATGTAGAACGCCAGT
OT2	crRNA: CCTTCTCAGAAGACCTACCCNGG<br>DNA: CCTTCTCAGGAGCCCTCCCCTGG	chr1:195746835-195746857	3	F: AAACACCTGCTATCTAAGCTG<br>R: CCCGAATACTCATGGTTGC
OT3	crRNA: CCTTCTCAGAAGACCTACCCNGG<br>DNA: CATTCTCAGCAGACCCACCCCGG	chr14:5758517-5758539	3	F: GCCCTCTCTGAATGAGTCTCGT<br>R: CCACCATCGGGAAAATACCAGT
OT4	crRNA: CCTTCTCAGAAGACCTACCCNGG<br>DNA: CCTGCTCAGAAGGCCTCCCCCGG	chr4:2993758-2993780	3	F: GTCCGTGGTCACCAGTGGTC<br>R: GCCACCTAAGTCTCACTGTTGC
OT5	crRNA: CCTTCTCAGAAGACCTACCCNGG<br>DNA: CCTTCCCAGAAGGCCTTCCCTGG	chr7:38386638-38386660	3	F: GCCCTATAAAGAGTTTAGCAT<br>R: TCTGTCACCTCTACATCCCC
OT6	crRNA: CCTTCTCAGAAGACCTACCCNGG<br>DNA: CCTTCTCTTAAGTCCTACCCAGG	chr7:43009897-43009919	3	F: TATATCCAGAGCCAACTAGTCA<br>R: TAGGCCCAGTCAATAACAGT
OT7	crRNA: CCTTCTCAGAAGACCTACCCNGG<br>DNA: CCTTCCCAGAAGACCCAGCCAGG	chr10:53780587-53780609	3	F: TGCACATCCTTTGTCAACTCA<br>R: TGCCTTTTCTTCCCTATGCTT
OT8	crRNA: CCTTCTCAGAAGACCTACCCNGG<br>DNA: TCTTCTCAGAAGCCCTTCCCAGG	chr11:74692873-74692895	3	F: GCTGCACATTTCATCATCCC<br>R: GGCCAGTAAACAAACATCACC
OT9	crRNA: CCTTCTCAGAAGACCTACCCNGG<br>DNA: CCTGCTCAGAAGCCCTGCCCTGG	chr17:39846377-39846399	3	F: CCCAGCACCTTTATACTCAC<br>R: GTGCAGTCAACAAAACACCATC
OT10	crRNA: CCTTCTCAGAAGACCTACCCNGG<br>DNA: GCTTCTCAAGAGAGCTACCCAGG	chr9:58847217-58847239	4	F: TGGCATTTCTCTGAACCAT<br>R: CCACCTAAGCCAGATGTTCC
